# Supplementary material for: Pyridoxamine Attenuates Doxorubicin-Induced Cardiomyopathy without Affecting Its Antitumor Effect on Rat Mammary Tumor Cells
Source: Cells. 2024 Jan 9;13(2):120. doi: 10.3390/cells13020120 (PMC10814382; doi:10.3390/cells13020120)
Supplement: Supplementary file 1 [file cells-13-00120-s001.zip › cells-2712842-supplementary.pdf]

Supplementary Tables

Table S1: Echocardiographic parameters of systolic LV function and volumes at baseline.

	**CTRL (N=14)**	**DOX (N=14)**	**DOX+PM (N=18)**	**CTRL+PM (N=14)**
LVEF (%)	82 ± 2	82 ± 1	82 ± 1	80 ± 2
LV cardiac index (mL/min/cm^2)	0.22 ± 0.01	0.24 ± 0.01	0.25 ± 0.01	0.25 ± 0.02
LVESV/BSA (µl/cm^2)	0.14 ± 0.02	0.15 ± 0.01	0.15 ± 0.01	0.16 ± 0.01
LVEDV/BSA (µl/cm^2)	0.78 ± 0.03	0.80 ± 0.03	0.84 ± 0.79	0.82 ± 0.74

The cardiac index was calculated as cardiac output (CO) normalized to body surface area (BSA). LVESV and LVEDV were also normalized to BSA. Data are presented as mean ± SEM. DOX, doxorubicin. LV, left ventricle. LVEF, left ventricular ejection fraction. LVEDV, left ventricular end-diastolic volume. LVESV, left ventricular end-systolic volume. PM, pyridoxamine.

Supplementary Figures

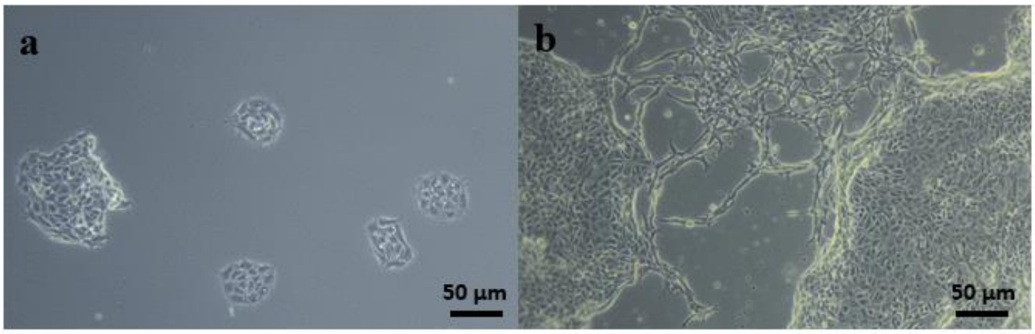

Figure S1: Morphology of LA7 mammary tumor cells. **a)** LA7 cells grown as a monolayer, characterized by a typical epithelial polygonal morphology. **b)** Branched-like structures one week after plating. Magnification: 20×.
